# Lack of affective priming indicates attitude-behaviour discrepancy for COVID-19 affiliated words

**DOI:** 10.1038/s41598-021-01210-9

**Published:** 2021-11-09

**Authors:** Stefania S. Moro, Jennifer K. E. Steeves

**Affiliations:** 1 Department of Psychology and Centre for Vision Research, York University, Toronto, Canada; 2 1032 Sherman Health Science Research Centre, York University, 4700 Keele St., Toronto, ON Canada

**Keywords:** Psychology, Human behaviour

## Abstract

The ongoing novel coronavirus (COVID-19) pandemic has resulted in the enforcement of national public health safety measures including precautionary behaviours such as border closures, movement restrictions, total or partial lockdowns, social distancing, and face mask mandates in order to reduce the spread of this disease. The current study uses affective priming, an indirect behavioural measure of implicit attitude, to evaluate COVID-19 attitudes. Explicitly, participants rated their overall risk perception associated with contracting COVID-19 significantly lower compared to their perception of necessary precautions and overall adherence to public health measures. During baseline trials, participants explicitly rated COVID-19 affiliated words as unpleasant, similar to traditional unpleasant word stimuli. Despite rating the COVID-19 affiliated words as unpleasant, affective priming was not observed for congruent prime-target COVID-19 affiliated word pairs when compared to congruent prime-target pleasant and unpleasant words. Overall, these results provide quantitative evidence that COVID-19 affiliated words do not invoke the same implicit attitude response as traditional pleasant and unpleasant word stimuli, despite conscious explicit rating of the COVID-19 words as unpleasant. This reduction in unpleasant attitude towards COVID-19 related words may contribute towards decreased fear-related behaviours and increased incidences of risky-behaviour facilitating the movement of the virus.

## Introduction

In late 2019 the novel coronavirus (COVID-19) outbreak was first detected and has since spread around the world with 164,523,894 confirmed cases and 3,412,032 COVID-19 related deaths across 222 countries, areas, and territories as of May 18, 2021^1^. Since there is no pharmaceutical cure the best way to mitigate this highly contagious and rapidly spreading virus is to prevent it from spreading^2–4^. Precautionary behaviors have been shown to help reduce the spread of infections through initiatives such as, quarantine of infected persons, social distancing through school and workplace closures, cancelation of large public gatherings, frequent handwashing, and the use of face masks^5,6^. Additionally, in areas that are undergoing a rapid increase in transmission, community-wide restrictions through lockdowns and stay-at-home orders have also been exercised in order to reduce the strain on the health care system^5,7^. These precautionary measures have proven effective for reducing the spread of viruses and contributed to mitigating the 1918 Influenza pandemic^8^, SARS in China^9,10^, Ebola in West Africa^11,12^, and Hepatitis E in South Sudan^13^.

Currently, precautionary behaviours, including closed borders, total or partial lockdowns, social distancing, movement restrictions, and face mask mandates have been adopted as the first line of defence to reduce the spread of COVID-19^14–16^. Emerging evidence implicates the employment of these types of precautionary behaviours as contributing to increasing the risk for pervasive mental health problems and psychological fear-related responses (for review^17,18^). Evidence for negative psychological responses to previous outbreaks, such as the Ebola Virus epidemic has been seen through increased fear-related behaviours, such as stigmatizing infected survivors and ignoring preventative medical procedures^19,20^. Furthermore, in approximately half of Ebola Virus survivors and their relations, widespread occurrence of anxiety, posttraumatic stress disorder, and depression has been observed^21,22^. High-risk behaviours such as, ignoring recommendations for social distancing (observed through sustained group gatherings) and continuing to travel despite restrictions have also been observed throughout the COVID-19 pandemic^22^. The onset of these high-risk behaviours contributes to accelerating the spread of the disease and makes it harder to contact trace and subsequently isolate suspected and confirmed cases^22^. Conversely, fear-related behaviours, such as extreme avoidance of social contact, contribute to increased risk of mental health problems^22^. This combination of high-risk and fear-induced behaviours shape the short and long-term trajectory of the outbreak^20,22,23^.

Human information processing and subsequent behaviours do not develop solely from the acquisition of knowledge but also from the surrounding environment where opportunities and risks must be evaluated^24^. This adaptive response where incoming stimuli are rated as pleasant or unpleasant, liked or disliked, good or bad occurs automatically, prior to conscience cognitive analysis of the stimulus^24^. The affective priming paradigm has been shown to be effective in illustrating this type of implicit evaluative response^24–26^. Affective priming investigates whether the assessment of a first stimulus (the prime) affects the processing of a subsequent stimulus (the target) (for review^24,27^). A facilitation effect emerges when a polarized target word (e.g. success) is preceded by a congruently-polarized prime word (e.g. diamond) rather than an incongruently-polarized prime word (e.g. torture) and leads to a faster response time^27^. The affective priming paradigm has emerged as an ideal indirect behavioural task that is able to probe into the dynamics of implicit evaluative processing^27^. It has been used in the context of evaluating racial attitudes^28–32^, health behaviour^33,34^, self-esteem^35,36^, food attitudes^37^, and clinical research issues^38,39^.

In the current study, through the observation of prime-target word pairs that belong to either pleasant or unpleasant affective categories we measure affective priming as an indirect behavioural measure aimed at evaluating implicit COVID-19 attitudes. Additionally, we directly measure COVID-19 attitudes through the COVID-19 Pandemic Mental Health Questionnaire (CoPaQ)^40,41^. We predicted that participants would perceive COVID-19 associated words as being unpleasant, and as a result would demonstrate affective priming similar to that expected with traditional pleasant and unpleasant words. Results from this study demonstrate whether simply associating COVID-19 words as unpleasant is sufficient to elicit an affective priming effect and thereby show an unconscious negative attitude toward COVID-19 associated words. This will provide a better understanding of how people cognitively process and interpret common COVID-19 associated words. Furthermore, results from this study will contribute to the growing body of research exploring why some individuals across communities might be more or less willing to engage in precautionary behaviours outlined by their public health agencies to mitigate the spread of COVID-19.

## Results

### COVID-19 attitudes

**Figure 1:**
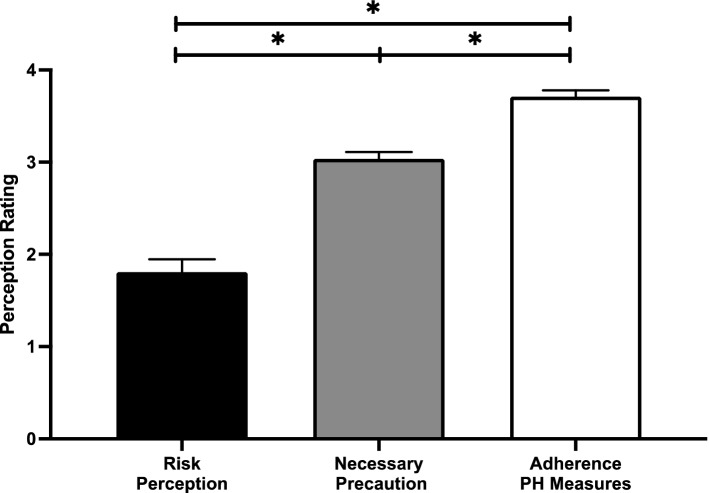
Self-reported rating on a 4-factor scale (where 4 indicates the greatest importance) for COVID-19 risk perception (black), perceived necessity for public health measures to mitigate COVID-19 (grey), and perception of an individual’s adherence to public health (PH) measures (white).

The assumption of homogeneity of variance was not met for these data and therefore we used Welch’s adjusted F ratio for this analysis. A one-way analysis of variance (ANOVA) comparing the overall rating for COVID-19 attitudes (risk perception, necessary precaution, and adherence to public health measures), was significant, *Welch’s F*(2, 96.742) = 79.178, *p* < 0.0001; ŋ^2^ = 0.552. Games-Howell corrected pairwise comparisons indicate that perceived risk perception was significantly lower compared to both the perception of the necessity for public health measures (*p* < 0.0001), and perceived adherence to public health measures (*p* < 0.0001). Additionally, the perceived necessity for public health measures is significantly lower than the reported perceived adherence to public health measures (*p* < 0.0001). Figure 1 plots the perceived risk, the perception of necessity for public health measures, and the perceived adherence to public health measures.

### Proportion of unpleasant responses

**Figure 2:**
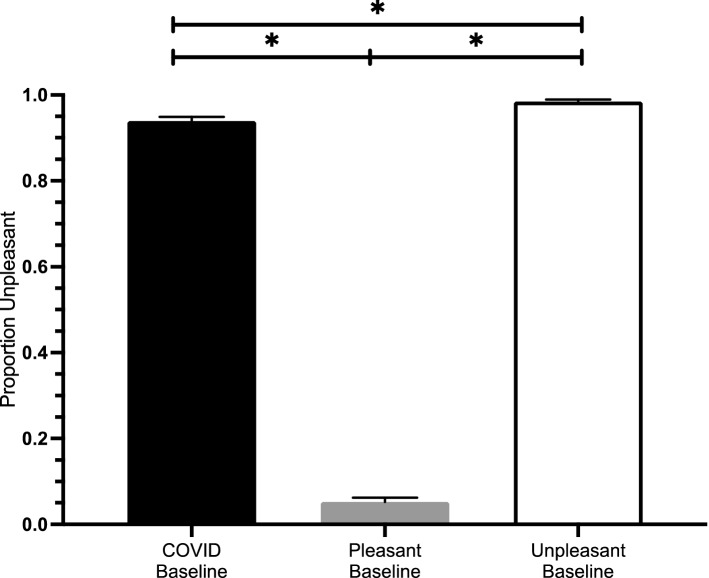
The proportion of unpleasant responses for each of the COVID-19 (black), pleasant (grey), and unpleasant (white) word categories. Both unpleasant and COVID-19 words were most often rated as unpleasant while pleasant words were rarely rated as unpleasant.

The assumption of homogeneity of variance was not met for these data and therefore we used Welch’s adjusted F ratio for this analysis. A one-way analysis of variance (ANOVA) comparing the proportion of unpleasant responses for each affect category (pleasant, unpleasant, and COVID-19) was significant, *Welch’s F*(2, 90.610) = 3600.26, *p* < 0.0001; ŋ^2^ = 0.981. Games-Howell corrected pairwise comparisons indicate that the proportion of unpleasant responses differed between the COVID-19 and pleasant categories (*p* < 0.0001), the COVID-19 and unpleasant categories (*p* < 0.0001) and the pleasant and unpleasant categories (*p* < 0.0001). Despite significant differences between proportion of unpleasant responses for each affect category, it is important to note that the COVID-19 affect category were consistently rated unpleasant (M = 0.94, SD = 0.07). Figure 2 plots the proportion unpleasant rated for each of the pleasant, unpleasant, and COVID-19 word categories.

### Reaction time

#### Baseline reaction time

**Figure 3:**
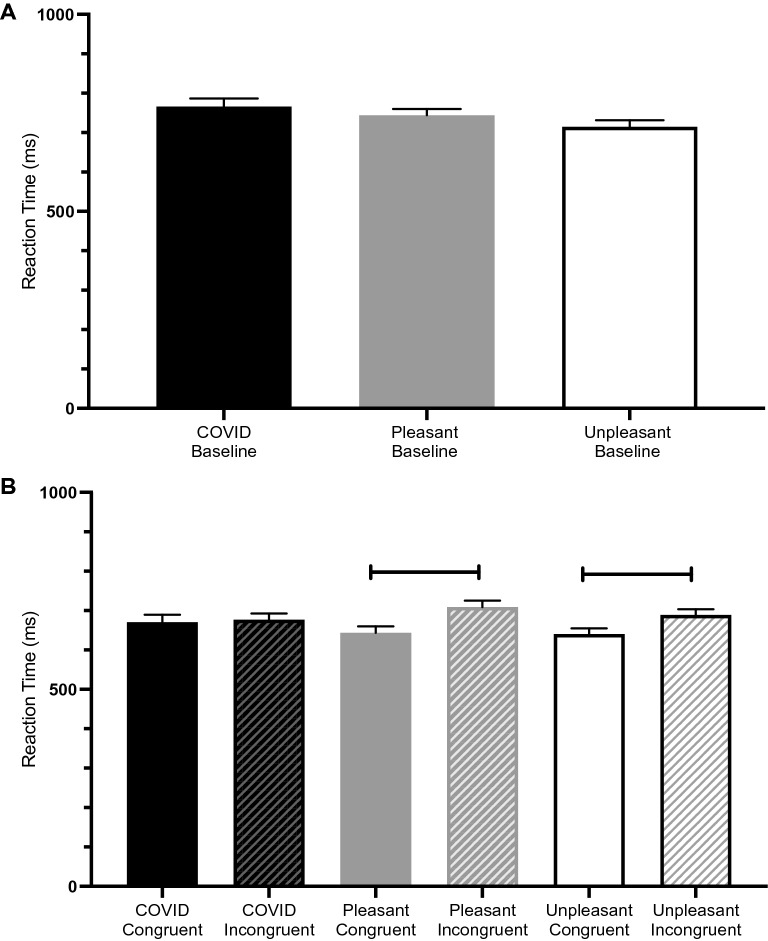
(**A**) The baseline reaction times for each of the COVID-19 (black), pleasant (grey), and unpleasant (white) word categories. (**B**) The congruent (solid) and incongruent (striped) prime-target reaction times for each of the COVID-19 pleasant and unpleasant word categories. Reaction times were shorter for congruent pleasant and congruent unpleasant word pairs, showing affective priming for these categories. There was no decrease in reaction time for COVID congruent word pairs.

A one-way analysis of variance (ANOVA) comparing the reaction time for baseline trials where each participant was presented with a string of asterisks (***) followed by every word from each affect category as a target was not significant, *F*(2, 155) = 2.105, *p* < 0.125; ŋ^2^ = 0.027. Figure 3A plots the reaction time for each of the baseline pleasant, unpleasant, and COVID-19 word categories.

#### Congruent and incongruent reaction time

We conducted a series of *t*-tests to investigate whether there was a difference in reaction time between congruent and incongruent prime-target pairings for each affective word category. There was no significant difference between congruent and incongruent reaction times for the COVID-19 word category, *t*(102) = -0.269, *p* = 0.788; *d* = 0.05. This indicates that there is no priming effect present for the COVID-19 word category. Congruent reaction times were significantly faster compared to incongruent reaction times for the pleasant word category, *t*(102) = -2.838, *p* = 0.005; *d* = 0.56, indicating a significant priming effect present for the pleasant word category. Finally, congruent reaction times were significantly faster compared to incongruent reaction times for the unpleasant word category, *t*(102) = -2.411, *p* = 0.018; *d* = 0.47, indicating a priming effect present for the unpleasant word category. Figure 3B plots the reaction time for each of the congruent and incongruent prime-target word pairs for the pleasant, unpleasant, and COVID-19 word categories.

### Difference scores

**Figure 4:**
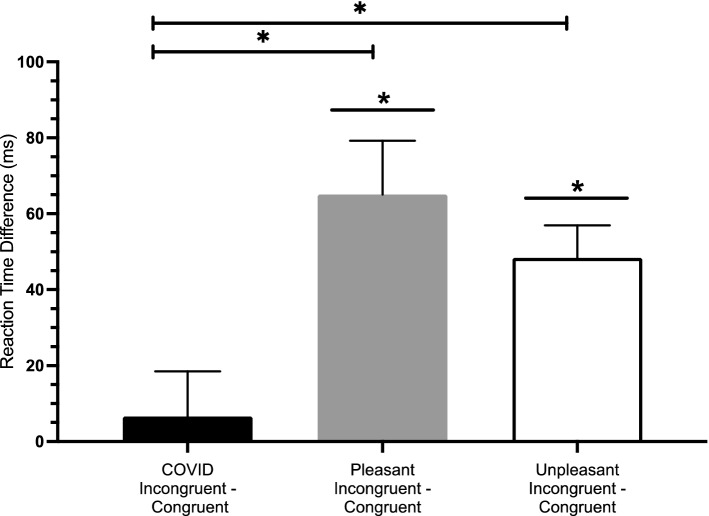
Difference scores, generated by subtracting congruent from incongruent reaction times, are plotted for each of the COVID-19 (black), pleasant (grey), and unpleasant (white) word categories. Pleasant (grey) and unpleasant (white) word category bias scores are also significantly different from zero indicating affective priming.

The assumption of homogeneity of variance was not met for these data and therefore we used Welch’s adjusted F ratio for this analysis. A one-way analysis of variance (ANOVA) comparing the difference scores generated by subtracting the congruent reaction time from the incongruent reaction time for each affect category (pleasant, unpleasant, and COVID-19) was significant, *Welch’s F*(2, 97.427 = 5.964, *p* = 0.004; ŋ^2^ = 0.08. Games-Howell corrected pairwise comparisons indicate that the COVID-19 difference scores were smaller compared to the pleasant (*p* < 0.006) and unpleasant categories (*p* < 0.014). There was no difference when comparing the pleasant and unpleasant categories (*p* = 0.573). Additionally, one sample *t*-tests were conducted to compare the mean difference score for each of the word categories to 0 in order to provide another analysis of the affective priming effect. COVID-19 words did not demonstrate a significant difference score compared to 0, *t*(51) = 0.556, *p* = 0.580; *d* = 0.077. Pleasant words demonstrated a significantly higher difference score compared to 0, *t*(51) = 4.596, *p* < 0.0001; *d* = 0.637, indicating a priming effect. Similarly, unpleasant words demonstrated a significantly higher difference score compared to 0, *t*(51) = 5.670, *p* < 0.0001; *d* = 0.786, indicating a priming effect. Figure 4 plots the difference scores (priming effect) for each of the pleasant, unpleasant, and COVID-19 word categories.

## Discussion

The current study used implicit affective priming as an indirect behavioural measure aimed at evaluating implicit COVID-19 attitudes. When asked explicitly, participants reported a significantly lower perception of risk associated with contracting COVID-19 compared to their perceived necessity of COVID-19 precautionary measures, as well as their perceived adherence to public health measures. During baseline trials of our priming task, participants rated COVID-19 affiliated words as unpleasant similar to traditional unpleasant word stimuli and unlike traditional pleasant word stimuli. Baseline reaction times to COVID-19 words did not differ compared to baseline reaction times to both pleasant and unpleasant indicating that word length and familiarity did not impact our results. Affective priming was observed for the pleasant and unpleasant prime conditions where the prime-target word pair were congruent compared to incongruent. Affective priming was not observed for the COVID-19 prime condition. Difference scores obtained by subtracting the congruent prime-target reaction time from the incongruent prime-target reaction time show the size of affective priming and were significantly larger for pleasant and unpleasant prime categories compared to the COVID-19 prime category. Overall, these results provide quantitative evidence that COVID-19 affiliated words do not invoke the same implicit attitude response as traditional pleasant and unpleasant word stimuli, despite explicit conscious rating of the COVID-19 words as unpleasant. These results align with our measure of COVID-19 attitudes indicating a decreased perception of risk.

Since the first observation of the affective priming effect by Fazio and colleagues (1986), there has been a growing body of research extending the use of this paradigm for investigation into the dynamics and mechanisms of evaluative processing^26^. Affective priming and other similar indirect behavioural measures such as the implicit association task^42^, the affective Simon task^43^, and the emotional Stroop task^44^ are frequently used as an indirect behavioural measure of attitude. Participant responses to target stimuli can be informative of the prime’s valence^37^. In a study investigating food likes and dislikes using the affective priming paradigm it was observed that by priming people with positive and negative food stimuli it was possible to assess food attitudes for both strongly and moderately evaluated food primes^37^. Our current results do not demonstrate an affective priming effect for COVID-19 related words, despite baseline evaluations of the words as unpleasant. The lack of affective priming in this case indicates that the participants in our study have not internalized their unpleasant attitude towards this word category.

Indirect implicit measures of attitude are important as they are less influenced by normative social demands and could potentially better predict behaviour in some circumstances^45^. These experimental designs permit attitudes to be measured experimentally from response patterns towards related stimuli without the influence of social normative pressures, which is not possible when directly asking participants^36^. This is especially important to consider in light of our current results since our baseline evaluations of the COVID-19 related words were strongly indicative of unpleasant attitudes that were not reflected during our implicit behavioural measure. Furthermore, when asked explicitly our participants demonstrated a significantly reduced perception of risk compared to both their report of the necessity of public health measures and their perceived adherence to public health measures. The present study’s data, collected at the peak of the third wave of the COVID-19 pandemic in Canada, confirm that participants may have been able to monitor their expression of normative attitudes^36^ to fall in line with prevailing public health messaging. The expression of normative unpleasant attitudes were not consistent for COVID-19 affiliated words compared to traditional unpleasant words for the affective priming trials which serve as an implicit indirect behavioural measure. These results are aligned with the decreased perception of risk observed through explicit questioning. Conversely, strong reports of adherence to public health measures obtained through the CoPaQ questionnaire is indicative of the influence of normative pressures rather than reflective of explicit or implicit COVID-19 risk perception.

An important secondary factor in interpreting our results relates to the mean age of participation in our group (28 years). It has been shown that through their increased movement within the community, young people, who often show limited to no COVID-19 symptoms despite being infected, facilitate the spread of the COVID-19 virus^46,47^. On average, young people have larger social networks^48^ and therefore a higher likelihood of participating in social behaviour during the pandemic^49^. Knowledge of the virus itself does not always lead to precautionary health behaviours but can be mediated by other factors such as risk perception^5,50–52^. A positive relationship between behaviour and risk perception was widely reported during the 2009 H1N1 influenza pandemic^53^. In light of these findings, our participants are likely not motivated by fear-related behaviours due to decreased severity of COVID-19 related illness in younger age groups which directly results in decreased fear internalization and an attitude-behaviour discrepancy towards COVID-19 related words^5^. Furthermore, it has been shown that increased fear behaviour towards the virus consistently predicts compliance with public health measures such as social distancing and hand hygiene^54^. It is possible that through this implicit behavioural measure our current results reflect the attitude-behaviour discrepancy that may result in an overall decrease in adherence to public health guidelines within this age group, despite their reports on the CoPaQ questionnaire.

Significant increases in the prevalence of mental health problems such as depression, anxiety, a combination of depression and anxiety, and posttraumatic stress disorder is widely reported throughout this time^55–58^. Mental health may have an impact on attitudes related to COVID-19 and subsequent behaviour. This is especially important to consider in our population as our younger participants may be at a higher risk of experiencing mental health problems during the current pandemic based on previous research indicating that they are more likely to experience higher levels of loneliness from social distancing measures, which is often a precursor to developing anxiety and depression^56^. Future large-scale studies investigating mental health and its relationship with behaviour through indirect measures across a wider age range of participants and geographical locations would contribute towards expanding upon the current results.

In conclusion, this study provides evidence of a reduction in unpleasant attitude towards COVID-19 related words. This may contribute towards decreased fear-related behaviours and increased incidence of risky-behaviour facilitating the spreading/movement of the virus. Overall, this study contributes to the growing body of research exploring why some individuals across communities might be more or less willing to engage in precautionary behaviours outlined by their public health agencies to mitigate the spread of COVID-19. Finally, this study indicates the potential for the affective priming paradigm as a useful quantitative measure to predict COVID-19 attitudes.

## Methods

### Participants

52 participants with a mean age of 28 years (SD = 4; 38 female) from York University in Toronto volunteered to participate in this study in exchange for course credit for an introductory psychology course (if applicable). All participants were self-described as native English speakers, and 3 participants were left hand dominant. Participants were excluded from this study if they did not report themselves as a native English speaker. All participants gave informed consent prior to their inclusion in the study, which was approved by York University Office of Research Ethics. All methods were performed in accordance with the York University Office of Research Ethics guidelines and regulations.

### Stimuli

Stimuli were made up of words from 3 affect categories: pleasant, unpleasant, and COVID-19. The pleasant and unpleasant word stimuli were selected from the list of pleasant and unpleasant words used in a previous study^29^. The pleasant and unpleasant word stimuli were originally taken from the Affective Norms for English Words (ANEW) that are rated based emotional valence and arousal^59^. Only high arousal words in the relevant emotional valence were selected for this study. The list of COVID-19 related words was selected from a broader list of 65 words that were top ranked according to strength of relationship to COVID-19 by 20 independent raters, where the average measure interclass correlation was 0.82 with a 95% confidence interval from 0.61 to 0.94 (F(10, 120) = 5.477, *p* < 0.001). 6 words from each affect category were selected for inclusion in the prime category and 5 different words from each affect category were selected for inclusion in the target category (see Appendix A). Additionally, there was no difference in word length across affective word categories (F(2, 30) = 0.5262, *p* = 0.596).

**Figure 5:**
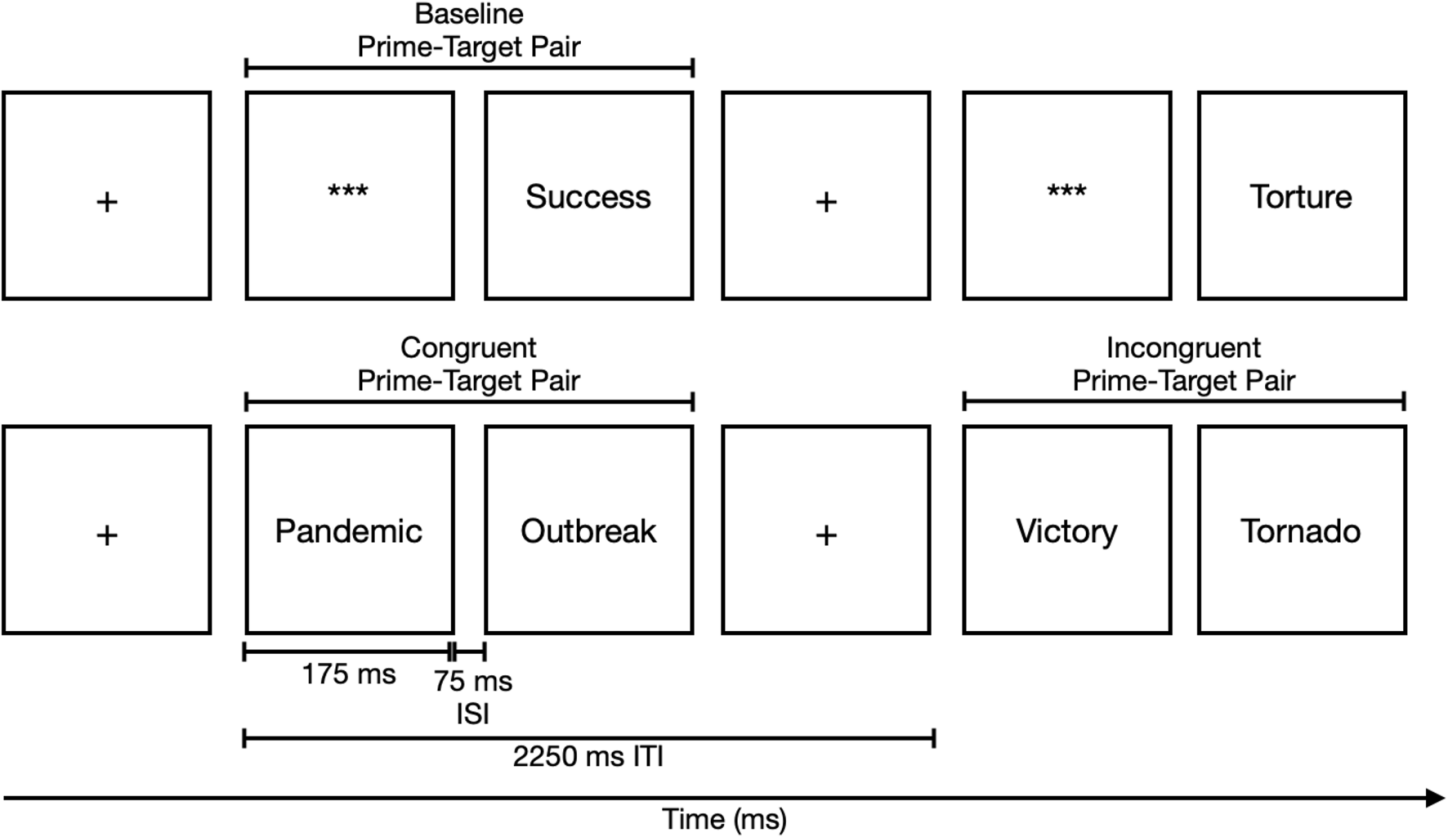
A schematic illustration of the presentation of affective word stimuli.

On each trial the affective valence of the prime and the target was either congruent or incongruent. There were 60 congruent trials where the prime and target were both pleasant, both unpleasant, or both COVID-19 related, with an equal amount of trials for each affective category. 60 incongruent trials where the prime was paired with a target that was not from the same affect category as the prime were included. Furthermore, 33 baseline trials where each word from each affect category was presented as a target paired with a string of asterisks (***) were also included and randomly presented intermixed with the congruent and incongruent trials. There were a total of 153 trials that were each 2250 ms in length, where the prime stimulus was presented for 175 ms followed by a 75 ms inter-stimulus interval and the target stimulus that was presented for the remaining 2000 ms or until the participant made a response (see Fig. 5). Reaction times were measured from the onset of the target.

### Procedure

Participants completed the entirety of this study online due to university restrictions to in-person participation because of the ongoing COVID-19 global pandemic. Qualtrics^60^ was used to administer the informed consent and demographic portion of this study and Inquisit^61^ was used for the affective priming portion.

Following the completion of the informed consent and demographic section, participants were asked to complete the COVID-19 Pandemic Mental Health Questionnaire (CoPaQ)^40,41^. The CoPaQ assesses COVID-19 contamination anxiety, countermeasure necessity and compliance, mental health impact, stressor impact, social media usage, interpersonal conflicts, paranoid ideations, institutional and political trust, conspiracy beliefs, and social cohesions (^40^). After completion of the questionnaire, participants proceeded with the affective priming experiment. Participants were asked to categorize affective target words into pleasant and unpleasant categories. Each participant completed 153 trials of randomly presented congruent and incongruent prime-target pairings, as well as baseline trials. Participants were instructed to respond as quickly and as accurately as possible by ignoring the first word (prime stimulus) and to classify the second word (target) as either pleasant or unpleasant using two corresponding buttons on their keyboard.

## Supplementary Information

Supplementary Information.
