# Supplementary material for: Lack of affective priming indicates attitude-behaviour discrepancy for COVID-19 affiliated words
Source: Sci Rep. 2021 Nov 9;11:21912. doi: 10.1038/s41598-021-01210-9 (PMC8578603; doi:10.1038/s41598-021-01210-9)
Supplement: Supplementary file 1 — Supplementary Information. [file 41598_2021_1210_MOESM1_ESM.pdf]

Lack of affective priming indicates attitude-behaviour discrepancy for COVID-19 affiliated words

Stefania S. Moro and Jennifer K. E. Steeves*
Department of Psychology and Centre for Vision Research, York University, Toronto, Canada

Supplementary Information

Appendix A

Affective Priming Words by Category

Pleasant Primes	*Pleasant Targets*	*Unpleasant Primes*	*Unpleasant Targets*	*COVID-19 Primes*	*Covid-19 Targets*
Triumph	Bouquet	Horror	Torture	Pandemic	Outbreak
Delight	Success	Traitor	Quarrel	Mask	Cases
Glory	Diamond	Hatred	Tornado	Lockdown	Isolation
Blossom	Passion	Misery	Deceit	Virus	Sanitizer
Victory	Fantasy	Prison	Robber	Quarantine	Fever
Miracle		Assault		Spread	
